# Knowledge of International Standards for Tuberculosis Care among Private Non-NTP Providers in Lagos, Nigeria: A Cross-Sectional Study

**DOI:** 10.3390/tropicalmed7080192

**Published:** 2022-08-18

**Authors:** Victor Abiola Adepoju, Kelechi Elizabeth Oladimeji, Olusola Adedeji Adejumo, Oluwatoyin Elizabeth Adepoju, Ademola Adelekan, Olanrewaju Oladimeji

**Affiliations:** 1 Department of HIV and Infectious Diseases, Jhpiego (An Affiliate of John Hopkins University), Abuja 900108, Nigeria; 2 Department of Public Health, Faculty of Health Sciences, Walter Sisulu University, Mthatha 5099, South Africa; 3 Lagos State University Teaching Hospital, Ikeja 101233, Nigeria; 4 Adolescents Friendly Research Initiative and Care (ADOLFRIC), Ado-Ekiti 360101, Nigeria; 5 Blue Gate Research Institute, Ibadan 200285, Nigeria

**Keywords:** private non-NTP providers, knowledge, tuberculosis, international standards for TB care

## Abstract

Studies specifically evaluating tuberculosis knowledge among private non-NTP providers using the International Standards for Tuberculosis Care (ISTC) framework are scarce. We evaluated the knowledge of ISTC among private non-NTP providers and associated factors in urban Lagos, Nigeria. We performed a cross-sectional descriptive study using a self-administered questionnaire to assess different aspects of tuberculosis management among 152 non-NTP providers in Lagos, Nigeria. The association between the dependent variable (knowledge) and independent variables (age, sex, qualifications, training and years of experience) was determined using multivariate logistic regression. Overall, the median knowledge score was 12 (52%, SD 3.8) and achieved by 47% of the participants. The highest knowledge score was in TB/HIV standards (67%) and the lowest was in the treatment standards (44%). On multivariate analysis, being female (OR 0.3, CI: 0.1–0.6, *p* < 0.0001) and being a nurse (OR 0.2, CI: 0.1–0.4, *p* < 0.0001) reduced the odds of having good TB knowledge score, while having previously managed ≥100 TB patients (OR 2.8, CI: 1.1–7.2, *p* = 0.028) increased the odds of having good TB knowledge. Gaps in the knowledge of ISTC among private non-NTP providers may result in substandard TB patient care. Specifically, gaps in knowledge of standard TB regimen combinations and Xpert MTB/RIF testing stood out. The present study provides evidence for tailored mentorship and TB education among nurses and female private non-NTP providers.

## 1. Introduction

Nigeria is a high burden country for tuberculosis (TB), tuberculosis/HIV co-infection and multi-drug resistance tuberculosis (MDR-TB) [1]. Despite the high burden of tuberculosis, only 26% (104,904) of the estimated 407,000 TB cases were reported to the National TB Program (NTP) in 2017, meaning that 74% or 300,096 cases were undetected or not reported to the NTP. Private providers contributed only 11% of TB cases notified in 2017 [2].

It was reported that the percentage of initial care seeking was 74%, 74%, 70%, 85%, 67%, 84% and 88% in India, Indonesia, Philippines, Pakistan, Nigeria, Bangladesh and Myanmar, respectively [3,4]. Private non-NTP providers are defined as private providers who are not part of the NTP surveillance, including qualified providers from the private for-profit (PFP) and private not-for-profit (PNFP) sectors. In Asia and Africa, countries such as Nigeria, India, China, Bangladesh, Pakistan, Indonesia and the Philippines have a high prevalence of TB and a huge private non-NTP facility volume. Oftentimes, care seekers prefer to access TB services in private non-NTP facilities due to their flexibility regarding diagnostic procedures, drug regimens and treatment observation methods, more convenient operating hours and locations, shorter waiting hours and lower administrative burden. Despite the large volume of patients visiting non-NTP facilities, a huge number of them remained unengaged by NTP in countries with a high presence of non-NTP providers. For instance, in Nigeria, despite the enormous patronage of private non-NTP providers, only 56% (277/406) of the PNFPs and 5% (646/13,448) of the PFPs were engaged by the NTP in 2017 [3]. There are concerns about the quality of TB care that private non-NTP facilities provide since they do not have access to TB capacity-building programs, policy document and guidelines, fixed-dose anti-TB medications and newer diagnostic infrastructures that are readily available in NTP-engaged private and public facilities.

Private non-NTP facilities might not follow local and international guidelines due to knowledge issues, with an inherent risk of over- and under-diagnosis of TB, as well as suboptimal treatment outcomes. In the cosmopolitan city of Bangalore, South India, 80% and 71% of private practitioners lacked adequate knowledge of TB diagnosis and TB treatment regimen, respectively. Moreover, private providers were reportedly unaware of the need for Directly Observed Therapy Short Course (DOTS) and follow-up sputum examination [5]. Similarly, in Nigeria, South Africa, Kenya and Tanzania, inadequate knowledge and non-compliance with National TB guidelines and policy recommendations were key barriers to effective TB management in the private sector [6,7,8]. Furthermore, critical knowledge gaps were reported regarding management of MDR-TB and TB/HIV co-infection [9,10,11]. Private practitioners in Pakistan had lower knowledge of TB diagnosis and management compared with physicians in public facilities, while Indian doctors failed to adhere to diagnostic and treatment standards as entrenched in the ISTC [12,13]. These findings could explain the conclusions made by a recent landscape analysis of private sector TB services by the World Health Organization (WHO) that inappropriate diagnoses among private providers was consequent upon poor knowledge of TB case management and lack of commitment to TB guideline implementation [14].

There are several barriers limiting efforts to engage private non-NTP providers. First, the model of engaging private non-NTP providers is largely donor-driven and relied on TB infrastructures and investments in the public sector. In the Philippines, for instance, while many private non-NTP providers acknowledged the benefits and values of engaging with the government NTP, such as access to training and knowledge exchange, free TB medications, reagents and reporting tools, they expressed dissatisfaction with the cumbersome engagement model that relied on public sector referrals for key TB diagnostics and sometimes prevention and treatment where many of these services were domiciled. Private providers highlighted the public sector’s limited operating hours and longer patient waiting times that oftentimes delayed the provision of TB services for private patients. Private providers also criticized steep requirements for paperwork as demoralizing and demotivating for NTO engagement [15]. Moreover, in Nigeria, some private providers were unaware of where and how to report and notify TB data to the public sector [16]. The aftermath was the poor quality of TB data and “missing TB” cases from the private non-NTP sector either because these TB cases were not diagnosed or notified to the NTP surveillance systems by the non-NTP providers [16].

Adequate clinician knowledge is critical to achieving appropriate regimen prescription and diagnostic investigation. Tuberculosis sensitization is one of the key activities toward the engagement of non-NTP providers to provide quality TB services in line with the NTP policy, the International Standard of TB Care (ISTC) and the Patient Charter as embedded in the National DOTS training curriculum [16]. Public–private mix (PPM) for TB prevention and care represents a comprehensive approach for a systematic involvement of all relevant health care providers in TB care to facilitate the use of International Standards for TB Care (ISTC) towards achieving the national TB target in the private sector. The effective roll-out of the PPM strategy could be further boosted through the International Standard for TB Care (ISTC). The ISTC is simple, less cumbersome and may be adapted to serve as a useful tool in securing a broad base of endorsements by private non-NTP providers, professional medical and nursing associations, academic institutions and non-governmental associations in high TB burden countries with considerable private sector presence. Simplification of the ISTC document is necessary to make the standards less theoretical and more practical. With rapid PPM expansion in Nigeria through donor investment in recent times, the investigators would like to establish baseline knowledge of ISTC standards and its predictors among private sector non-NTP providers. It is hoped that the outcome of this study will guide future guidelines and tools for continuous monitoring of the quality of TB care among private non-NTP facilities and providers.

## 2. Materials and Methods

### 2.1. Study Design

We performed a cross-sectional descriptive study using self-administered questionnaires to assess the knowledge of ISTC among 152 private non-NTP providers across 13 Local Government Areas in Lagos, Nigeria. The study took place between May 2018 and January 2019.

### 2.2. Setting

The study was conducted in Lagos, Nigeria. Lagos state has a population of over 20 million and is divided into 20 LGAs [17]. Lagos harbors 11% of the Nigerian population and has the highest burden of tuberculosis. The state is divided into 20 Local Government Areas (LGAs), and an LGA TB supervisor is responsible for the supervision of each LGA. Healthcare service in Lagos is provided at 3 levels: primary, secondary and tertiary. Every private doctor or nurse registers with the regulatory agency in Lagos before its operation. The Health Facility Monitoring and Accreditation Agency (HEFAMAA) is a parastatal agency in the Lagos State Ministry of Health responsible for the registration, supervision, monitoring as well as accreditation of private facilities. Over 3500 private clinics have been registered in Lagos to date. HEFAMAA uses a supervisory checklist to routinely monitor the quality of care provided by all private and public hospitals in Lagos. However, the tools to assess processes and practices are not necessarily TB-specific.

Lagos State TB and Leprosy Control Program (LSTBLCP) was inaugurated in 2003 and expanded to engage the private sector in 2008. Facilities are often engaged under four service schemes, i.e., referral of presumptive TB only, provision of Directly Observed Therapy Short Course (DOTS) only, provision of diagnosis service only and provision of both diagnosis and DOTS [18]. Since 2015, GeneXpert has been adopted as the gold standard for the diagnosis of TB in Nigeria while microscopy was the mainstay of follow-up when on TB treatment. Diagnosis of TB could also be made by collecting 2 sputum samples (one spot, one early morning) for microscopy when GeneXpert is not available, especially in rural areas. Despite the registration of several private providers (for-profit and not-for-profit) with respective associations and HEFAMAA, many of them were neither engaged with nor notified TB cases to NTP surveillance system, meaning that most managed TB cases evaded the National TB Program and WHO surveillance. In 2019, only about one-quarter of the over 3500 registered private clinics in Lagos were engaged by NTP.

In this study, 13 high-burden LGAs (Alimosho, Ifako-ijaiye, Ajeromi, Ojo, Badagry, Kosofe, Apapa, Agege, Mushin, Ikeja, Oshodi, Amuwo-odofin, Shomolu) were purposively selected from 20 LGAs in Lagos based on existing pilot PPM project implementation. Participants were selected and recruited between May 2018 and January 2019 through the various quarterly LGA meetings of private sector associations of doctors and nurses, i.e., the Association of General Private Medical Practitioners of Nigeria (AGPMPN) and the Association of General Private Nursing Practitioners (AGPMP). In addition, the Lagos TB program was contacted for a list of private clinics and nursing and maternity homes that were already engaged for TB notification. Facilities on this list were then excluded from the list shared by the AGPMN and AGPMP since the focus of the study was the private non-NTP engaged providers. Questionnaires were administered to assess the baseline knowledge of the invited private non-NTP engaged providers.

### 2.3. Study Size

A total of 152 providers from private non-NTP engaged facilities were included in the study. We calculated sample size using the formula n = a^2^b/d^2^, where n = sample size, a = Z statistic for a level of confidence, b = prevalence and d = precision or confidence interval. The level of confidence of 95% is conventional at which the value for a is 1.96 and d is 0.05. Dosumu (2008) previously estimated the prevalence of knowledge of tuberculosis DOTS among private providers in Nigeria as 9.8% [19]. This equates to a b value of 0.098. We arrived at an approximate sample size of 152 participants. With an assumption of a 20% non-response rate, a total of 180 participants were invited to participate in the study.

### 2.4. Sampling Technique

A 4-stage sampling technique (summarized below) was used to select participants for this study.

#### 2.4.1. Stage 1: Selection of Study LGAs

The purposive sampling technique was used to select 13 LGAs with a high TB burden and private sector presence in Lagos, Nigeria.

#### 2.4.2. Stage 2: Selection of Private Providers

The TB facilities were stratified into private NTP engaged and private non-NTP engaged and a convenient sampling technique was used to select the private non-NTP engaged facilities from the focused LGAs.

#### 2.4.3. Stage 3: Selection of Private Non-NTP Facilities

All the engaged private facilities were filtered from the universal list of all private facilities by cross-checking the NTP list with those of HEFAMMA and private sector associations. A systematic random sampling approach was used to select the required numbers of assessment private non-NTP facilities per LGA, in a representative sample of LGAs in the state, using www.randomizer.org (accessed on 5 July 2022) to generate random numbers from the list of private unengaged facilities in each of the 13 LGAs.

#### 2.4.4. Stage 4: Selection of Providers from the Non-NTP Engaged Private Facilities

The purposive sampling technique was used in selecting the required numbers of health care providers (doctors and nurses) interviewed in the selected health facilities. The medical director (a doctor) was selected for the private medical facility, while the nursing director was selected for private nursing homes. Where the medical director or nursing director was not available in a facility, they were replaced by a representative doctor or nurse who met the inclusion criteria. If more than one health worker met the inclusion criteria, simple random balloting was then used to select respondents to be interviewed.

### 2.5. Study Participants

Participants were private sector doctors and nurses (hospital owners) who were not yet engaged by the National TB and Leprosy Control Program to provide TB services but unofficially manage TB patients and report to the Integrated Disease Surveillance and Response (IDSR) systems. These include doctors and nurses from hospitals as well as nursing and maternity homes.

### 2.6. Inclusion and Exclusion Criteria

**Figure 1:**
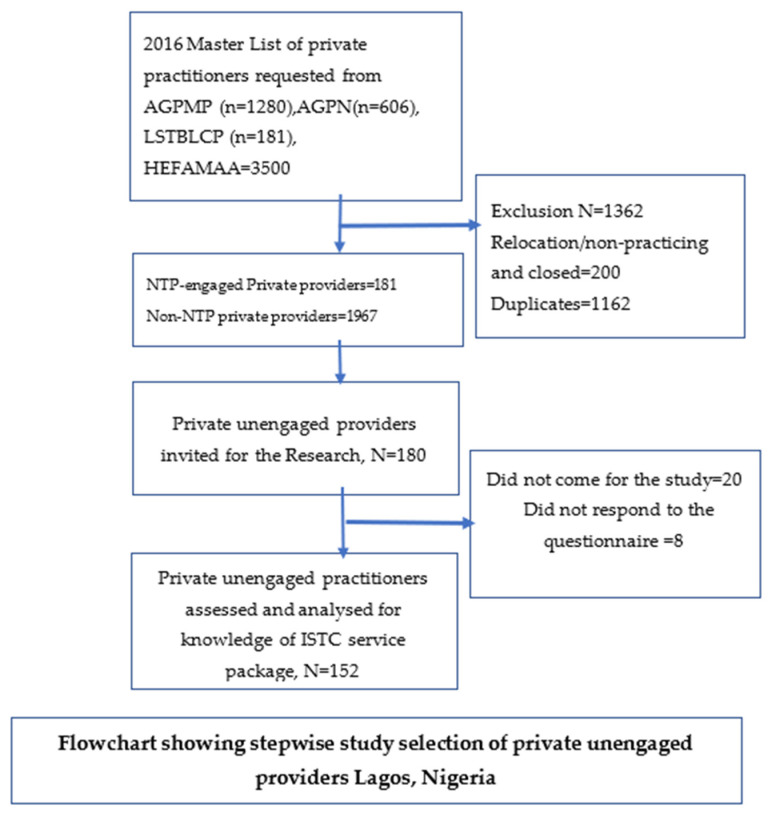
Flowchart showing stepwise study selection of private unengaged providers Lagos, Nigeria. HEFAMAA—Health Facility Monitoring and Accreditation Agency, AGPMPN—Association of General Private Medical Practitioners of Nigeria, AGPN—Association of General Private Nurses, LSTBLCP—Lagos State Tuberculosis, Buruli Ulcer and Leprosy Control Program.

To be included in this study, the provider must own, manage or co-manage a private practice (private hospital, nursing or maternity home), not engaged by the National TB Program, be aged 18 years or more and have diagnosed at least 1 TB case in the past. Private providers who were already trained and engaged by the National TB Program to notify TB, who never diagnosed any TB case in the past or who were younger than 18 years were excluded from the study (Figure 1).

### 2.7. Variables

The outcome variable was the ISTC knowledge score, categorized into good and poor knowledge depending on whether the score was above or below the median score. Independent variables include participants’ age, sex, practice setting, qualification, previous TB training, number of post-graduation years of experience and awareness of DOTS and DOTS facilities. Study variables were presented using descriptive statistics such as frequency and percentage. Knowledge score was categorized as good and poor knowledge composite variables based on the median score. Associations with good knowledge scores were assessed at a multivariate level using logistic regression.

### 2.8. Data Sources/Measurement

The 3rd edition of ISTC, published in 2014, was adapted and modified to align with the domestic requirements of TB guidelines in Nigeria [20]. The English version of the instrument was retrieved. English was also the language used to modify the instrument for this study. These standards (screening/diagnosis, treatment, TB/HIV co-infection and public health standards) were modified by a team of international and national experts. The modification builds on the 3rd edition published in 2014 and integrated information based on the national guidelines, new evidence and data. The modification also relied on the recent WHO guidelines, policy recommendations and peer-reviewed scientific literature produced after the 3rd edition was published in 2014. The 21 standards of the 3rd edition were assessed and each standard was further reworded into smaller micro-standards. After 2 rounds of meetings, a consensus was reached between the experts and the Lagos State TB Program until the final document was approved. This document provides up-to-date standards for tuberculosis care tailored to Nigeria setting and based on the recently published policy statements and international guidelines. Some of the changes made to the original standards include the age of IPT eligibility for children and the duration and combinations of DR-TB treatment among others. The instrument was validated with a Cronbach alpha score of 0.89.

The questionnaire for this study was developed according to these modified standards to serve as an instrument for baseline assessment of TB knowledge among these private non-NTP providers. The standards on public health were further sub-divided into a new section on human rights for emphasis on this theme. The study questionnaire comprises two sections. Section one collects the socio-demographics and other details regarding the participants’ practice setting, qualification and years of experience in tuberculosis management. Section two contains 30 multiple-choice questions that assessed the knowledge of the participants with a different number of questions across the five standards categories based on the International Standard for TB Care TB. Standards assessed include knowledge of symptom and diagnosis (7), knowledge of treatment (10), knowledge of management of TB/HIV co-morbidity (2), knowledge of public health (7) as well as the TB patient rights charter (4).

The correct response to each question was assigned a score of “1” and the incorrect response was assigned a score of “0”. Questions with no responses were scored as incorrect (0). The total score was obtained by summing up a score for each of the 30 questions either as ISTC overall score or performance of individual standard. Participants’ score below the median score was classified as having poor knowledge and those who scored the median and above the median score were classified as having good knowledge. Poor and good knowledge scores were ultimately coded as 0 and 1, respectively, after conversion into categorical variables. The proportion of the correct responses in each standard category is descriptively described and discussed here. The proportion of participants who fell under the poor and good knowledge categories is also presented.

The questions were pilot tested before the start of the study in ten selected non-NTP private facilities that were not part of the study. Questionnaires were self-administered after extensive explanation to the participants. Participants were pre-instructed and provided additional clarifications about the questions where needed. Questionnaires were also checked for completeness after they were returned by the participants. Independently trained data entry clerks entered the data into Microsoft Excel before they were imported into Statistical Package for Social Sciences (SPSS) version 17 for further analysis.

### 2.9. Bias

In order to minimize bias, the study comprises private non-NTP facilities across 13 LGAs from both urban and semi-urban populations, as well as large and small-sized facilities.

### 2.10. Data Collection

Data were collected by six trained data clerks using a Microsoft Excel data collection template that was developed for this study. In minimizing bias in data extraction, data clerks were trained on the data collection processes. The research was also piloted outside the study setting to test the practical knowledge of data collection by the trained data clerks. The objective of the study was also masked from the data clerks. Daily reviews were held with data collectors to assess collected data for any missing information or double counting. No double counting was observed during the process. Before analysis, collected data by data clerks were triangulated between the hard copies of the questionnaire and what had been entered into the data entry template. Collected data were also randomly picked by multiple observers and approved by team supervisors to ensure interrater reliability. For each respondent, data were collected on age, sex, awareness of TB/DOTS services and facilities providing them, awareness of NTP, monthly OPD attendance, qualification, years of clinical practice, prior participation in TB-specific training and the last TB training attended and having previously managed TB patients. Information collected on the characteristics of the non-NTP engaged facilities includes the type of health facility (for-profit or not-for-profit) and monthly OPD attendance.

### 2.11. Data Analysis

Responses were first entered into Microsoft Excel, then checked, cleaned and imported into Statistical Software for Social Science (SPSS) version 17 for coding, categorization and statistical analyses. In addressing the research objective, we applied descriptive statistics (percentages and numbers) to summarize the categorical variables—socio-demographic status—and described the knowledge of ISTC (poor or good). Mean and SD were used to describe normally distributed continuous variables. A bivariate logistic regression model was first fitted and the variables which had a *p* value < 0.05 in the bivariate analysis were fitted in the final multivariable logistic regression model. Variables with a *p* value < 0.05 in the final multivariable logistic regression model were considered significantly associated with the dependent/outcome variable (i.e., knowledge of ISTC). Crude and adjusted ORs with 95% CI were calculated to measure the strength of association between the dependent/outcome (knowledge of ISTC) and independent variables (gender, type of health facility, awareness of NTP, previous management of TB cases, prior TB training, primary qualification, number of TB patients managed in the past, number of years of postgraduate study, OPD attendance). On bivariate analysis, gender, number of TB patients managed in the past, qualification of private provider, prior TB training and type of facilities were significant predictors of good ISTC knowledge, but only gender, provider qualification and number of TB patients managed in the past became significant predictors of good knowledge of ISTC on multivariate analysis.

## 3. Results

Only 160 of the 180 invited providers came for the study. The non-response rate was 8/160 (5%). All the remaining 152 questionnaires were completed. Table 1 shows the demographic characteristics and private non-NTP providers’ level of awareness and knowledge of ISTC. The majority were 20–34 years old (36.8%), female (58.6%), operate PFP (91.4%), have heard about DOTS 116 (76.3%), were not aware of DOTS facilities (61.2%), were aware of NTP (65.8%), were qualified nurse/midwives (60.5%), had 1–16 years of practice experience (53.3%) and never had TB-specific training (69.7%).

Table 2 shows the proportion of private non-NTP providers with correct and incorrect responses to each of the ISTC knowledge questions. The most failed question was Q6, i.e., “How many sputum samples are needed for the diagnosis of TB using microscopy?” About 140/152 (92.1%) of the private non-NTP providers wrongly stated that three sputum samples or more were required to diagnose TB with microscopy. In Q3, 66.4% did not know that GeneXpert was the standard test to diagnose TB in Nigeria, while in Q10, 67.1% of them wrongly stated that GeneXpert or clinical judgment was required to monitor TB treatment.

Table 3 shows how private non-NTP providers performed across the various ISTC standards of care. Overall, the median knowledge score was 12 (52%, SD 3.8). For each standard category, the highest median score was obtained in TB/HIV standards (67%), while the lowest median score was obtained in the treatment standards category (44%).

Table 4 shows the ISTC knowledge scores and associated factors. On multivariate analysis, having good knowledge of ISTC was significantly associated with gender, professional qualification and number of previously managed TB patients. Being female (OR 0.3, CI 0.1–0.6, *p* < 0.0001) and being a nurse private practitioner (OR 0.2, CI 0.1–0.4, *p* < 0.0001) reduced the odds of having a good TB knowledge score, while having previously managed >100 TB patients (OR 2.8, CI 1.1–7.2, *p* = 0.028) increased the odds of having good TB knowledge.

## 4. Discussion

The objective of the study was to determine the knowledge of the International Standards of TB Care among private doctors and nurses who were not engaged with the National TB program in Lagos, Nigeria.

The most failed question by the majority (92.1%) was the number of samples needed to diagnose TB. Moreover, multiple sputum AFB samples were requested for TB diagnosis, while most participants relied on GeneXpert and clinical judgement to monitor TB treatment. These findings are similar to outcomes of studies that assessed the quality of TB diagnosis in India, Ethiopia and Nigeria [21,22,23,24,25]. This underscores the failure of private non-NTP providers to keep track of updates in TB diagnostic approaches. Requesting multiple samples for diagnostic microscopy increases the risk of DR-TB spread and pre-diagnostic loss to follow up from the economic burden of repeat patient visits. Relying on GeneXpert to monitor TB treatment has enormous implications when assigning TB treatment outcomes for patients who were initially bacteriological positive but monitored radiologically. Patients monitored by GeneXpert could be subject to a prolonged duration of treatment emanating from a false impression of non-conversion of GeneXpert-detected dead bacilli. Training and supervision should emphasize these knowledge gaps.

The median ISTC knowledge score in this study was 12 (52%). This finding is comparable with the 52% median ISTC score observed among doctors and nurses during the 2016 Hajj [26], lower than the 67.3% (10/15) observed in Lima, Peru but higher than the 14% (28/201) and 10.5–48% reported from India and Pakistan respectively [11,12,13]. It is difficult to compare many of these studies with our findings because of the small sample size and inclusion of other healthcare workers such as laboratory technicians, community health workers and administrative staff. Moreover, some of the studies neither mentioned the study setting (private or public) nor the NTP engagement status of the facilities. However, healthcare workers in studies focusing on public hospitals or involving providers who combined both public and private practices appeared to have better knowledge scores, possibly because of the exposure to training materials in the public sector. This confirmed the previous finding that public doctors have better knowledge of TB compared with private doctors [12].

Shockingly, recent TB training was not associated with a good knowledge score, despite the majority being trained recently. This could be because only 30% were trained in our study and most of the training for non-NTP providers was usually a few hours of TB sensitization, which might not be comprehensive enough to impact the knowledge score. The observed “knowledge–action gap”, described as inconsistencies in private non-NTP providers’ ability to translate knowledge gained from training into practice, results in ineffective TB prevention and control in private non-NTP facilities. A study among private providers in South Africa quantified the know–do gaps—the difference between GPs’ intended and observed practices—using standardized patients, and clinical vignettes observed know–do gaps of 37% for TB and 18% for HIV [27]. Therefore, the emphasis should be on the application and translation of knowledge gained from routine NTP training and periodic supervision. As staff mobility is rampant in the private sector, TB programs should also build an incentive system to attract and retain staff in TB control programs towards sustainable investment in knowledge and capacity building, as recommended by health providers in the Dominican Republic [28].

In our study, those who managed over 100 TB patients in the past had good knowledge of ISTC compared with those who managed less. This echoes findings in Pakistan, where providers who saw more than 12 patients with TB per year had significantly higher median ISTC scores than those who saw fewer patients with TB [12]. Rather than accumulated years of practice, it appears from our study the importance of TB-specific case management experience in boosting knowledge. This unfortunately is often lacking among many small-scale private non-NTP providers. For instance, in Nigeria, a review noted that 30% of PFP clinics and 12% of PNFP clinics registered no TB cases, while 6% and 16% PNFPs and PFPs, respectively, registered only 1 TB case in 2017 [3]. Demand creation activities such as community tuberculosis screening outreaches and the public to private sector referral of TB patients based on proximity to patients should be encouraged among non-NTP providers. Treating TB patients regularly offers a more practical opportunity to assess competency- and scenario-based learning.

Nurses had significantly lower odds of good TB knowledge in this study compared with doctors. This is similar to the finding from San Juan, Peru [11], but different from the report from India, which found no statistically significant difference between the TB knowledge score of MBBS doctors and other types of providers [29]. This may be because the scenario-based vignette methodology used in the India study was more difficult as it required knowledge application, unlike the multiple-choice styled question format in the current study. In the Philippines, private non-NTP engaged nurses wanted NTP to improve the utilization of available communication channels and keep them informed on the latest research and TB policy recommendations [15]. Platforms such as continuous medical education (CME) programs have been successfully used as the leading source of TB knowledge for both public and private sector doctors and nurses in Pakistan. The representation of professional associations in NTP training and further step-down through private sector professional representatives have been suggested as a cost-effective approach to implementing this model [12]. A recent systematic review also suggested leveraging private sector professional associations to expand engagement with non-NTP providers of TB services [30]. Therefore, partnership with various training colleges and professional associations of nurses and doctors to include TB-specific updates in pre-service curriculum and continuous professional development programs is an effective strategy to keep private non-NTP providers abreast of updates in rapidly changing tuberculosis programs.

Women had significantly lower odds of good TB knowledge compared with men in this study. This is similar to findings from South Africa [31] and Southwest Ethiopia [32] but different from another study from South Africa where women had better TB knowledge scores than men [33]. The gender dimension of tuberculosis knowledge in the study reflects similar differentials in doctors’ and nurses’ TB knowledge since many nurses were also females. Many of these women own maternity and nursing homes and would oftentimes attend to pregnant and vulnerable elderly patients who might also be at risk of tuberculosis. Female non-NTP engaged private providers could be vital in tuberculosis detection among pregnant and breastfeeding women. They need to be prioritized in tuberculosis control efforts, including capacity building and mentorship opportunities.

### Limitations

The study has some strengths and limitations. First, the findings of the study must be interpreted and generalized with caution since the study took place in Lagos, where the quality of health services is generally better than realities elsewhere in the country. Furthermore, the study is not representative of all private non-NTP providers, as informal retail pharmacies were not included in this study. However, this study can serve as a model for similar intermediate and high TB burden countries that share national priorities for strengthening knowledge of tuberculosis among private non-NTP providers as part of the TB control efforts. The completion rate of the present study was good (95%) and only 5% of study participants did not respond to the questionnaire. The study questionnaire was developed based on the ISTC 3rd edition disseminated by the Curry TB Center and further modified by TB experts, making it a useful and reliable tool to assess TB knowledge of private non-NTP providers. The study also lacked a comparison control group but will serve as a baseline against which future provider knowledge can be measured and compared. Furthermore, additional variables that influence the knowledge of non-NTP providers, such as dual practice, duration and type of previous TB training and source of training, were not collected. For instance, some providers either currently operate in both government and private practice or had previously worked in government hospitals and were possibly exposed to NTP training resources, which might have influenced their general knowledge of TB. The cross-sectional design of this survey does not allow the evaluation of causal relationships, but it does make associations between several factors that represent, in any case, the basis for testing causal hypotheses in further studies. Our survey represents a good starting point for future longitudinal studies aimed at assessing the impact of changes in the knowledge of private non-NTP providers post-engagement. Despite the limitations, the study findings have implications for policy and practice. The result establishes baseline knowledge of ISTC standards among private non-NTP providers, and it is the first in recent times to assess provider knowledge using the ISTC framework in sub-Saharan Africa.

## 5. Conclusions

The surveyed private non-NTP providers had inadequate knowledge levels of TB, as measured by the International Standards for Tuberculosis Care, particularly regarding TB treatment standards with negative implications on TB treatment outcome, the spread of DR-TB and overall TB control efforts in high TB burden countries. Some critical knowledge gaps were observed in the areas of TB case detection and management. This survey also underlines the importance of clinical experience and regular encounters with TB patients in clinical practice as a veritable pathway towards competency-based learning by doing. For small-scale private providers or those operating in settings with low TB prevalence, a referral system that will shift the burden of TB caseload from high burden public and private facilities to these smaller scale/low patient load private facilities should be considered. Community outreaches as part of the corporate social responsibility of the private non-NTP facilities could help them to maintain quality diagnosis and treatment of TB patients. We suggest that intern nurses and doctors should also rotate through the TB and HIV clinics and practically consult a minimum prescribed number of TB patients during medical internship. The poorer knowledge observed in this study among nurses also draws attention to why improvement interventions should prioritize these groups of professionals. For instance, engaging the professional accreditation institutions for the inclusion of TB and HIV modules as part of the CME program or pre-service curriculum will provide a platform for programmatic TB updates. At the level of the professional health associations, a medical representative model where a representative could participate regularly in NTP update training and step down to the membership may also be effective from a cost and policy making perspective, considering that TB is a dynamic area with new data emerging regularly. Overall, much work is necessary to improve the quality of TB care in the private non-NTP sector. Initiatives such as the ISTC need to be combined with dissemination and translational efforts of pilot projects at the country level, coupled with robust measurement approaches (e.g., standardized patients) to see whether standards can change practice and improve patient outcomes. National TB programs will therefore need to systematically measure and improve the quality of TB care and invest in quality improvement programs.

## Figures and Tables

**Table 1:** Demographic characteristics and participants’ level of awareness and knowledge of TB.

Variable	Frequency	Percent
**Age**		
20–34	56	36.8
35–49	46	30.3
50–64	40	26.3
>65	10	6.6
**Sex**		
Male	63	41.4
Female	89	58.6
**Type of health facility**		
Private Not-For-Profit	13	8.6
Private For-Profit	139	91.4
**Heard about TB/DOTS before**		
NO	36	23.7
YES	116	76.3
**Aware of TB DOTS facilities in your area**		
NO	93	61.2
YES	59	38.8
**Aware of NTBLCP**		
NO	52	34.2
YES	100	65.8
**Monthly OPD attendance**		
<100	57	37.5
100–200	46	30.3
200–500	33	21.7
>500	16	10.5
**Qualification**		
MBBS/GP	60	39.5
NURSE/MIDWIVE	92	60.5
**Years of clinical practice**		
1–16	73	53.3
17–44	64	46.7
**Previously managed TB patient**		
NO	61	40.1
YES	91	59.9
**Prior participation in TB-specific training**		
NO	106	69.7
YES	46	30.3
**Last participation in TB training (years ago)**		
<1	9	19.6
1–5	18	39.1
5–10	14	30.4
>10	5	10.9

NTBLCP—National TB, Leprosy and Buruli Ulcer Control Program; GP—general practitioner; MBBS—Bachelor of Medicine and Surgery; DOTS—Directly Observed Therapy Short course.

**Table 2:** Participants’ responses to questions assessing knowledge of tuberculosis diagnosis, treatment, TB/HIV co-infection, public health and patient rights.

	Standard 1 (TB Diagnosis)		Correct Answer (%)	Incorrect Answer (%)
1		Which of the following tests requires a blood sample for the diagnosis of TB	42 (27.6)	110 (72.4)
2		TB is most frequently caused by	139 (91.4)	13 (8.6)
3		The standard test for diagnosis of tuberculosis in Nigeria is	51 (33.6)	101 (66.4)
4		Where are the missing TB cases	100 (65.8)	52 (34.2)
5		Which of the following is a symptom of tuberculosis	115 (75.7)	37 (24.3)
6		How many sputum samples are needed for the diagnosis of TB using microscopy	12 (7.9)	140 (92.1)
7		Which is TRUE concerning TB disease	66 (43.4)	86 (56.6)
	**Standards 2 (TB Treatment)**			
8		Traditional treatment for drug-resistant TB lasts for how many months	16 (10.5)	136 (89.5)
9		Drug-sensitive TB is treated for how many months	111 (73.0)	41 (27.0)
10		Which of the following tests should be used to monitor the success of treatment	50 (32.9)	102 (67.1)
11		TB treatment success is a combination of	114 (75.0)	38 (25.0)
12		Which of these drugs is not a first-line anti-TB drug currently in use for the treatment of TB	76 (50)	76 (50.0)
13		Concerning drug-resistant TB	83 (54.6)	69 (45.4)
14		Which of the following is a possible treatment outcome for a TB patient on treatment	119 (78.3)	33 (21.2)
15		What is the full meaning of the commonly used acronym DOTS	60 (39.5)	92 (60.5)
16		Which of the following information is mandatory before initiating TB medication	73 (48)	79 (52)
17		Which of the following is INCORRECT about DOTS	64 (42.1)	88 (57.9)
	**Standards 3 (Management of TB/HIV Co-infection)**			
18		Which of the following interventions will lower the risk of active TB in people living with HIV (PLHIV)	47 (30.9)	105 (69.1)
19		Which of the following is not a component of the 3Is of TBHIV collaboration	84 (55.3)	68 (44.7)
	**Standards 4 (Public Health and Contact Investigation)**			
20		Which of these high-risk populations should be targeted for latent tuberculosis treatment	74 (48.7)	78 (51.3)
21		TB can be spread typically through the following means	46 (30.3)	106 (69.7)
22		The highest priority contacts for evaluation are	82 (53.9)	70 (46.1)
23		Which groups are considered more likely to be exposed to or infected with M. Tuberculosis	107 (70.4)	45 (29.6)
24		This is not true of latent TB infection (LTBI) and TB disease	39 (46.4)	45 (53.6)
25		What is the preferred prophylaxis for the prevention of TB in eligible adults	51 (33.6)	101 (66.4)
26		How to stop tuberculosis	117 (77.0)	35 (23.0)
	**Standards 5 (TB Patient Right)**			
27		Concerning patient rights, which is CORRECT among the following	110 (72.4)	42 (27.6)
28		Which of the following is CORRECT concerning patient-centered care (PCC) versus the medical model of care	36 (23.7)	116 (76.3)
29		The rights of TB patients include the following except	62 (40.8)	90 (59.2)
30		In TB, program PCTC implies	33 (21.7)	119 (78.3)

ISTC—International Standards for Tuberculosis Care; DOTS—Directly Observed Therapy Short Course; PCC—patient-centered care; PLHIV—people living with HIV.

**Table 3:** Aggregate knowledge score of participants on tuberculosis standards of care.

TB Knowledge Area	Median Score (%)	Range	S.D.
Diagnosis score	3.0(60)	0–5	1.3
Treatment score	4.0(44)	0–9	1.9
TB/HIV	2.0(67)	0–3	0.7
Public health	2.0(50)	0–4	1.6
Patient rights	1.0(50)	0–2	0.9
Overall knowledge score	12.0(52)	0–23	3.8

**Table 4:** Factors associated with total knowledge of tuberculosis among private non-NTP engaged providers in Lagos, Nigeria.

Variable	Poor Knowledge (<12)	Good Knowledge (>12)	OR, 95% CI	*p*-Value
**Gender**				
Male	22 (27.5)	41 (56.9)	Ref.	
Female	58 (72.5)	31 (43.1)	0.3 (0.1–0.6)	<0.0001 *
Age				
<35	26 (32.5)	30(41.7)	0.7 (0.3–1.3)	0.242
>35	54 (67.5)	42 (58.3)		
**Type of facility**				
PNFP	9 (11.3)	4 (5.6)	2.2 (0.6–7.3)	0.21
PFP	71 (88.8)	68 (94.4)		
**Awareness of NTP**				
Yes	27 (33.8)	24 (34.7)	0.9 (0.5–1.9	0.9
No	53 (66.3)	47 (65.3)		
**Previously managed TB Patient**				
No	34 (42.5)	27 (37.5)	1.2 (0.6–2.4)	0.53
Yes	46 (57.5)	45 (62.5)		
**Prior TB training**				
No	56 (70)	50 (69.4)	1.0 (0.5–2.0)	0.941
Yes	24 (30)	22 (30.6)		
**Primary qualification**				
MBBS/GP	17 (21.3)	43 (59.7)	Ref.	
Nurse	63 (78.8)	29 (40.3)	0.2 (0.1–0.4)	<0.0001 *
**No. of TB patients managed before**				
<10	19 (41.3)	9 (20)	Ref.	
10–100	27 (58.7)	36 (80)	2.8 (1.1–7.2)	0.028 *
**No. of years postgraduate**				
1–16 years	32 (50)	38 (57.6)	0.7 (0.4–1.5)	0.386
17–44 years	32 (50)	28 (42.4)		
**OPD attendance**				
<100	35 (43.8)	22 (30.6)	1.8 (0.9–3.4)	0.093
>100	45 (56.3)	50 (65.4)		

* Significant *p*-value < 0.05.

## Data Availability

The data presented in this study are available on request from the corresponding author. The data are not publicly available due to privacy restrictions.

## Abbreviations

ISTC	International Standards of TB Care
CME	Continuous medical education
NTP	National TB Program
DR-TB	Drug-resistant TB
TB	Tuberculosis
PFP	Private for-profit
PFNP	Private not-for-profit
HIV	Human immunodeficiency virus
MBBS	Bachelor of Medicine and Surgery
GP	General practitioner
OPD	Outpatient department
DOTS	Directly Observed Therapy Short Course
AGPMP	Association of General and Private Medical Practitioners of Nigeria
SPSS	Statistical Software for Social Sciences
HEFAMAA	Health Facility Monitoring and Accreditation Agency
LSTBLCP	Lagos State Tuberculosis Buruli Ulcer and Leprosy Control Program
IDSR	Integrated Disease Surveillance and Response
LGA	Local Government Area
WHO	World Health Organization
